# Casticin protected against neuronal injury and inhibited the TLR4/NF‐κB pathway after middle cerebral artery occlusion in rats

**DOI:** 10.1002/prp2.752

**Published:** 2021-03-11

**Authors:** Dan Huang, Jiafan Zhou, Wenning Li, Li Zhang, Xiaomeng Wang, Qiang Liu

**Affiliations:** ^1^ Department of Neurology Hainan General Hospital Hainan Affiliated Hospital of Hainan Medical University Haikou China; ^2^ Department of Neurology Qionghai People's Hospital Qionghai China; ^3^ Department of Pharmacology Hainan Medical University Haikou China

**Keywords:** casticin, neuroprotective, stroke, TLR4/NF‐κB pathway

## Abstract

Although stroke is a major human neurological disease, there is a paucity of effective neuroprotectants that can improve its treatment. Casticin is a natural monomer drug with many biological effects such as anti‐inflammatory and anti‐tumor actions. However, it is not clear whether it has a neuroprotective effect in ischemic stroke. In this study, the neuroprotective effect of casticin in a rat middle cerebral artery occlusion (MCAO) model was investigated. Results showed that casticin reduced the volume of the cerebral infarction, mNSS scores, swimming distance, time to find the submerged platform, and serum concentrations of TNF‐α, TGF‐β, IL‐6 in MCAO rats. Moreover, casticin also decreased the expression of TLR4, NF‐κB p65, and NF‐κB p50 proteins and reversed the reduced expression of IκB protein in the brain tissue of MCAO rats. The *in vitro* study revealed that casticin decreased apoptosis of OGD/R‐PC12 cells, reduced the expression of TLR4, NF‐κB p65, and NF‐κB p50, while increased IκB protein expression. In conclusion, casticin improved the neurological functions of MCAO rats via inhibiting the TLR4/NF‐κB pathway and might have the potential to be developed into a neuroprotective agent for stroke patients.

## Graphical abstract

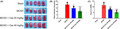

## 1 INTRODUCTION

Stroke is a common neurological disease. With high morbidity, relapse, mortality and disability rate, it is known as one of the three leading causes of death. The majority of strokes (about 80 percent) are ischemic in nature.^1^, ^2^, ^3^ At present time, tissue plasminogen activator (tPA) is the only treatment approved by the FDA for ischemic stroke. However, treatment with tPA has a strict time window and <5% of ischemic stroke patients receive tPA.^4^ Neuroprotective therapy could be an important approach for early treatment of stroke, which is a biochemical process that affects the ischemic cascade responses and further blocks the destruction of neurons.^5^ Therefore, it is an important strategy to search for neuroprotective drugs to improve the success rate of stroke therapy.

Toll‐like receptor 4 (TLR4) is a transmembrane receptor protein of the innate immune system and belongs to the type I transmembrane glycoprotein receptor family.^6^ It is widely expressed in various cell types in the central nervous system, especially microglia and astrocytes.^7^ During the cerebral ischemia‐reperfusion process, endogenous ligands activate nuclear factor‐κB (NF‐κB) through the TLR4 signaling pathway, and induce the production of a large number of proinflammatory factors, chemokines, adhesion molecules, and other molecules to produce inflammatory cascades that exacerbate brain tissue damage.^8^ Studies have shown that under the condition of cerebral ischemia or cerebral ischemia‐reperfusion, TLR4 gene‐knockout mice had significantly reduced infarct areas and volumes, decreased activation of NF‐κB and expression of inflammatory factors and related mediators, compared with wild‐type mice.^9^, ^10^ In addition, studies have found that the number of TLR4‐single cells in peripheral blood increased significantly in patients with cerebral ischemia, and the expression of TLR4 was closely related to the degree of inflammation detected after stroke.^11^ These findings indicate that the TLR4‐mediated inflammatory response pathway is involved in the cerebral ischemic injury, and is expected to become an important therapeutic target for the prevention and treatment of cerebral ischemic stroke.

Casticin is a flavonoid extracted from *Rhizoma butensis*, which has various biological effects such as anti‐tumor, anti‐inflammatory, and improvement of airway responses in asthma.^12^, ^13^, ^14^ Besides, it has remarkable effects on nuclear factor, erythroid 2‐like 2 (Nrf2), kelch‐like ECH‐associated protein 1 (Keap1), NF‐κB, and other targets of oxidative stress and inflammation.^15^ To provide a new perspective for neuroprotective therapy of ischemic stroke patients, the present study explored the neuroprotective effects and the potential mechanism of action of casticin on middle cerebral artery occlusion (MCAO) of rat brain and on cultured oxygen glucose deprivation/reperfusion (OGD/R) PC12 cells, and focused on the TLR4/NF‐κB inflammatory pathway.

## 2 MATERIALS AND METHODS

### 2.1 Experimental animals and drug administration

**FIGURE 1:**
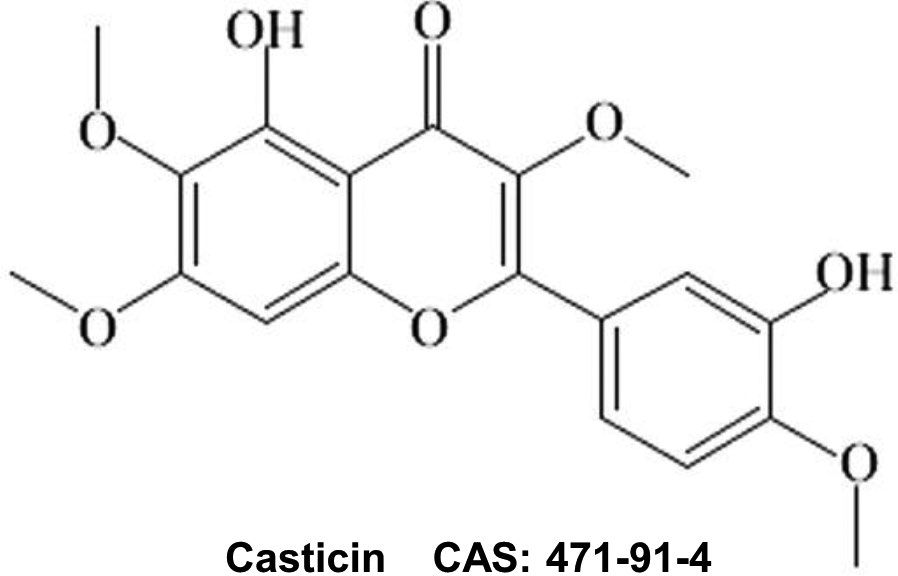
Chemical structure of casticin

A total of 40 male Sprague–Dawley rats (Shanghai Alac Laboratory Animal Co. Ltd, Shanghai, China, weighing between 220 and 260 g, Qualified number: 20170005003621) were randomly divided into four groups including Sham, MCAO, MCAO +casticin 20 mg/kg, and MCAO +casticin 40 mg/kg (10 rats in each group). The experimental groups received oral administration of casticin (Chengdu Herbpurify Co. Ltd, whose purity was ≥98%, Chengdu, China) of 20 mg/kg or 40 mg/kg, whereas the Sham‐operation group and the MCAO group received sterile distilled water at the same volume daily. Casticin was dissolved in sterile distilled water and administrated by gavage. The MCAO rats were served as control group and administrated sterile distilled water by gavage. The chemical structure of casticin is shown in Figure 1.

**FIGURE 2:**
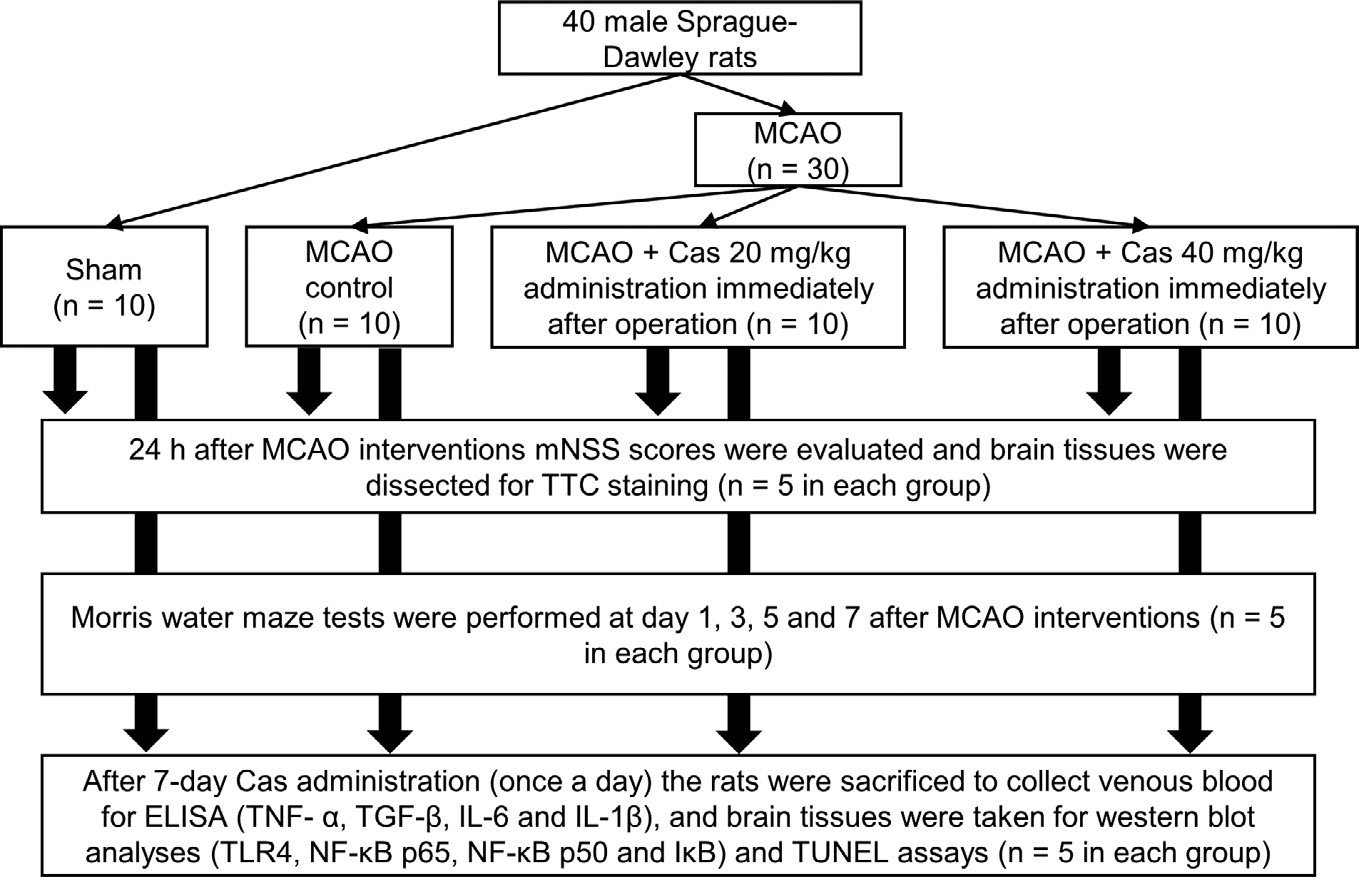
Flowchart of the MCAO experiments

All rats were fed standard rodent chow and sterilized secondary ultra‐pure water. In animals were kept in the feeding environment of a light and dark cycle for 12 hours, ambient temperature 22–25°C, and a humidity of 40%–70%. The experimental environment was employed for 7 days. The animal ethics committee of Hainan Medical University approved all experimental procedures conducted on rodents. Each MCAO rat received casticin immediately after operation, among which five rats were randomly killed and brain tissues were obtained for 2, 3, 5‐triphenyltetrazolium chloride (TTC) staining and modified neurological severity score (mNSS) determinations 24 hours after the MCAO intervention. The remaining five MCAO rats received casticin continuously for 7 days (once a day). Another 10 rats served as the MCAO controls (administrated with sterile distilled water by gavage) and 10 untreated rats were assigned to the Sham group (Figure 2).

### 2.2 MCAO model

A rat model of focal cerebral ischemia‐reperfusion injury was established using Koizumi's method.^16^ Rats were anesthetized by intraperitoneal injection of 10% chloral hydrate (300 mg/kg) (Sigma–Aldrich, CA, US). After supine fixation, skin preparation, and routine disinfection of the neck, a median incision was made to separate the right common carotid artery, external carotid artery, and internal carotid artery. Then, the distal end of the external carotid artery was ligated and a temporary clamp applied to the proximal end of the common carotid artery and the internal carotid artery. A small incision was made in the external carotid artery near the bifurcation of the common carotid artery, and a special monofilament coated with silica gel was inserted into one end. After that the vessel clip on the internal carotid artery was loosened and the monofilament gently inserted from the external carotid artery into the internal carotid artery until the monofilament blocked the beginning of the middle cerebral artery. The depth of the monofilament ranged from 18 to 20 mm. Subsequently, the cord was tightened, the clamp on the common carotid artery loosened, and the neck skin sutured. After ischemia for 2 hours, the monofilament was removed to restore blood flow reperfusion. In the sham group, only the internal carotid artery was isolated without any treatment. After the model was established, 100,000 units of penicillin sodium (Sigma‐Aldrich, CA, US) were injected for 3 days to prevent bacterial infection.

### 2.3 Neurological function test

At 24 hours after MCAO treatment, the neurological activity of the MCAO rats in each group was measured using the modified Neurological Severity Scores (mNSS) to assess motor, sensory, reflex, and balance functions. The total possible score was 18 and an mNSS score of 0 indicates normal. The higher the mNSS score the more serious the neurological damage.^17^

### 2.4 TTC staining

At 24 hours after MCAO treatment, the rats were anesthetized by intraperitoneal injection of 10% chloral hydrate (300 mg/kg) and the thorax and auricula dextra were cut open. After perfusion of PBS from the left ventricle, the whole brain was taken and frozen. Then, five coronal slices from the frontal pole were cut in 1.5‐mm‐thick sections and incubated in 2% TTC (Sigma‐Aldrich, CA, US) solution at 37°C for 30 minutes. The slices were then fixed in 4% formaldehyde for 24 hours before the determination of the infarct area. A high resolution pathological image analysis system (Champion Image HPIAS‐1000, Champion Image Engineering Company of Tongji Medical University, Wuhan, China) was used to measure the area of cerebral infarction in each region, and the infarct volume was calculated according to the formula V = t (A_1_ +…+ A_n_) – (A_1_ + A_n_) t/2, where T is the section thickness and A the infarct area.

### 2.5 Morris water maze testing

Morris water maze tests were conducted to evaluate the spatial learning ability of rats. The circular pool (120 cm in diameter, 50 cm in height, 30 cm water in depth, 22 ± 1°C water temperature) was placed in an independent lightproof laboratory. The pool was divided into four quadrants (E: east, S: south, W: west, N: north). Before the MCAO initiation, each rat was trained twice a day for 120 s/time for 3 days. On the 1st, 3rd, 5th, and 7th day after MCAO treatments, the positioning navigation experiment was conducted. The platform was placed in any quadrant and located 2 cm underwater. Adjacent and opposite quadrants of the platform were selected as entry points to determine the experimental incubation period (from entering the water to finding the platform) and the swimming distance. The determination scheme was carried out in accordance with the instructions of the manufacturer (SuperMaze Morris water maze experimental analysis system, Shanghai XinRuan Information Technology Co., Ltd.).

### 2.6 Enzyme‐linked immunosorbent assay (ELISA)

Venous blood was collected from the lower abdominal cavity and left static for 2 hours at room temperature. The serum was collected after centrifugation at 3000 rpm for 10 minutes at 4°C. The expression levels of tumor necrosis factor‐α (TNF‐α), transforming growth factor‐β (TGF‐β), interleukin 6 (IL‐6), and interleukin‐1β (IL‐1β) in the serum were measured according to the instructions of the ELISA kit (R&D Systems, Minneapolis, US) and ELISA (SpectraMaxM4, MD Company, US) kit, at a wavelength of 570 nm.

### 2.7 TdT‐mediated dUTP‐biotin nick end labeling (TUNEL)

Rats were deeply anesthetized by intraperitoneal injection of 10% chloral hydrate (300 mg/kg, *vide supra*), and then their heads were severed. The brains were cut in half according to the ischemic core. Half was used for Western Blotting assay. Half was fixed in 4% paraformaldehyde for 4 days. Then the brain tissue was embedded in paraffin and sectioned into 4 µm slices. Five slices in the ischemic core were obtained. The sections were dehydrated in alcohol gradients and incubated with 20 μg/ml protease K without DNase (Beyotime Biotechnology, Shanghai, China) at 37°C for 30 minutes. Subsequently, the samples were washed with PBS three times and were then ready for TUNEL staining (according to the procedure of TUNEL kit, Beyotime Biotechnology, Shanghai, China). Slides were re‐stained with 4’,6‐diamidino‐2‐phenylindole (DAPI) (Beyotime Biotechnology, Shanghai, China) for 10 minutes, and imaged under a fluorescence microscope to evaluate the degree of apoptosis. Five random images in one slice were obtained and the mean TUNEL positive cell number from five images was calculated as the TUNEL positive cell number of one rat. The images were obtained and analyzed by a staff blinded to the group setting.

### 2.8 Cell culture and OGD/R

PC12 cells were purchased from the Institute of Biochemistry and Cell Biology at the Chinese Academy of Sciences (Shanghai, China) and cultured in Dulbecco's modification of Eagle's medium (DMEM) with 10% fetal bovine serum and 5% penicillin–streptomycin (Gibco, NY, US) at 37°C in a humidified atmosphere containing 5% CO_2_.

OGD/R‐PC12 cells were established following the guidelines of Huang et al^18^ and pretreated with nerve growth factor (NGF) at a concentration of 50 µg/L for 48 hours. Then the PC12 cells were cultured for 24 hours in a complete medium and then washed twice with sugar‐free DMEM followed by incubation with sugar‐free DMEM for 2 hours in a closed anoxic chamber with the atmosphere of 5% CO_2_ and 95% N_2_ at 37°C. The cells that grown in a complete medium containing 10 µM casticin were incubated in an incubator with 5% CO_2_ at a constant temperature of 37°C for 24 h.

### 2.9 Flow cytometry assay

After 24 hours of casticin (10 µM) treatment, OGD/R‐PC12 cells were collected by centrifugation and resuspended in a 500 µl 1× binding buffer. To the cell suspension, 5 μl Annexin V‐FITC was added, followed by 10 μl PI (Annexin V‐FITC Detection Kit, Beyotime Biotechnology, Shanghai, China), which was mixed and then incubated at 4°C for 10 minutes under light‐free conditions. The cells were then detected by a flow cytometer (Accuri C6, Becton‐Dickinson, US).

### 2.10 Western blot analysis

OGD/R‐PC12 cells were collected or cortical brain tissue was isolated from the ischemic core and mixed with RIPA lysis buffer (Beyotime Biotechnology, Shanghai, China) to lyse cells or tissue blocks. Then samples were centrifuged at 14,000 rpm at 4°C for 30 minutes to collect the supernatant as the total protein. A BCA protein assay kit (Beyotime Biotechnology, Shanghai, China) was used to determine the protein concentration in the samples. The protein was subjected to electrophoresis on 10% sodium dodecyl sulfate‐polyacrylamide gel (SDS‐PAGE) and transferred onto polyvinylidene fluoride (PVDF) membranes (Millipore, MA, US). The blotting membranes were then incubated with 5% skim milk for 2 hours and washed with TBST buffer three times. Subsequently, the samples were incubated with anti‐NF‐κB p65 (ab16502), anti‐NF‐κB p50 (ab32360, ab28849), anti‐NF‐κB inhibitor protein (IκB)‐α (ab109300), and anti‐TLR4 (ab217274) antibodies with anti‐β‐actin antibodies (ab179467) as the internal control (1:1000, Abcam, Cambridge, MA, US) overnight at 4°C and then washed with TBST buffer three times. IgG (1:2,000, MultiSciences, Shanghai, China) was added for 1 hour at room temperature, and then specimens were washed with TBST buffer three times. An enhanced chemiluminescent (ECL) kit (Beyotime Biotechnology, Shanghai, China) was used for color development, and bands were detected by the chemiluminescent imaging system (ChemiDoc XRS + System, Bio‐RAD, California, US) and analyzed by Quantity One.

### 2.11 Nomenclature of targets and ligands

Key protein targets and ligands in this article are hyperlinked to corresponding entries in http://www.guidetopharmacology.org, the common portal for data from the IUPHAR/BPS Guide to PHARMACOLOGY,^19^ and are permanently archived in the Concise Guide to PHARMACOLOGY 2019/20.^20^

### 2.12 Data analysis

Data from five rats were collected independently for statistical analysis in each experiment, and the data are presented as the mean ± SD. GraphPad Prism 6.0 software was used for all statistical analyses. One‐way analysis of variance (ANOVA) was used followed by Bonferroni post‐hoc test correction for more than two conditions and then Dunnett's test for multiple comparisons to interpret the differences between three or more groups. Two‐way ANOVA was used followed by Dunnett's multiple comparisons test for Morris water maze testing. A *P*‐value <0.05 was considered to be a statistically significant finding.

## 3 RESULTS

### 3.1 Casticin improved animal functional recovery and reduced the cerebral infarction area in MCAO rats

**FIGURE 3:**
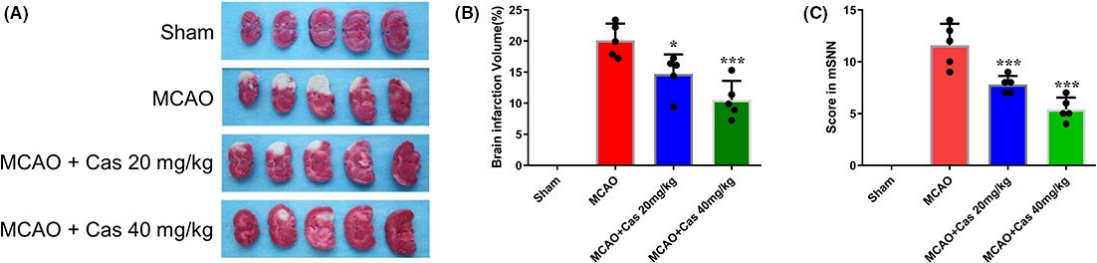
Cerebral infarct volume in MCAO rats 24 h after the MCAO treatment. (A) Cerebral infarction volume (white areas) in specimen and (B) Results of TTC staining (n = 5); (C) Effect of casticin on mNSS scores in MCAO rats (n = 5). *p < 0.05, ***p < 0.001 compared with the MCAO group. Cas, casticin; MCAO, middle cerebral artery occlusion

TTC staining was used to estimate the severity of the cerebral infarction. The rats were treated with casticin for 24 hours after MCAO modeling. TTC staining results showed that the volume of the cerebral infarction in 20 mg/kg and 40 mg/kg casticin treatment groups was 14.70 ± 3.14 (%) and 10.53 ± 3.06 (%), respectively, both of which were significantly lower than in the MCAO group (20.11 ± 2.68 (%), Figure 3A,B). In addition, the differences of cerebral infarction volumes between the 20 mg/kg casticin and 40 mg/kg casticin MCAO groups were also reflected in higher mNSS scores in the 40 mg/kg casticin rats 24 hours after the MCAO interventions (Figure 3C).

**FIGURE 4:**
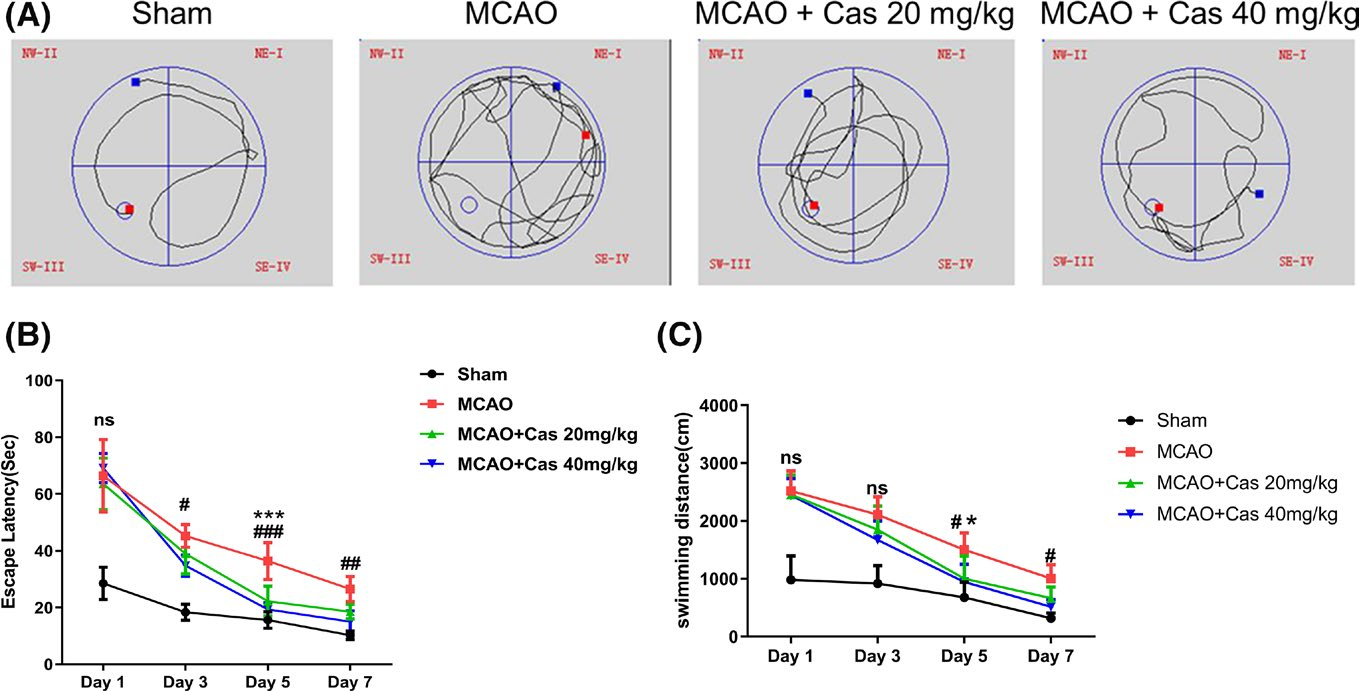
Neurological functions in MCAO rats measured by Morris water maze testing at day 1, 3, 5, and 7 after MCAO modeling. (A) Movement trajectory of the rats in each group (NE‐I: first quadrant, NW‐II: second quadrant, SW‐III: third quadrant, SE‐IV: fourth quadrant, red square is the starting point, and blue square is the ending point); (B) the incubation period (s) to find the platform within 2 min (n = 5); (C) the swimming distance (cm) to find the platform within 2 min (n = 5). * MCAO group compared with MCAO +Cas 20 mg/kg group; #, MCAO group compared with MCAO +Cas 40 mg/kg group;ns, no significance; *p < 0.05, **p < 0.01, ***p < 0.001

Next, the results of Morris water maze tests to evaluate the spatial learning ability and neurological function of the rats are shown in Figure 4. The escape latency of the rats in 20 mg/kg and 40 mg/kg casticin treatment groups was less compared with the MCAO group, and the swimming distance to find the platform was also shorter, suggesting that the learning ability of rats was significantly improved after casticin treatment. Taken together, the data demonstrated that casticin could effectively reduce the area of cerebral infarction in MCAO rats and improve the neurological functions and spatial learning ability.

### 3.2 Casticin reduced the production of inflammatory cytokines in MCAO rats

**FIGURE 5:**
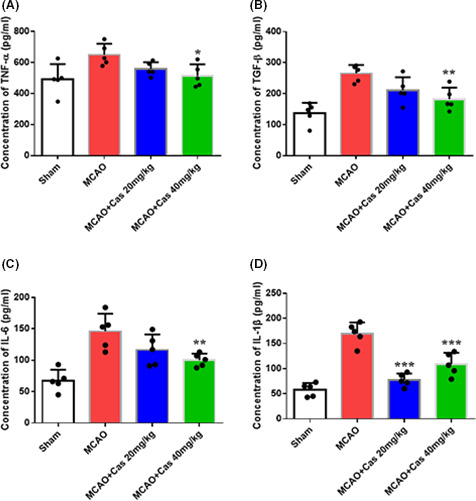
The expression levels (pg/mL) of TNF‐α, TGF‐β, IL‐6, and IL‐1β in rat serum 7 days after MCAO modeling were measured with ELISA (n = 5 for each group/condition). *p < 0.05, **p < 0.01, ***p < 0.001. IL‐1β, interleukin‐1β; IL‐6, Interleukin 6; TGF‐β, transforming growth factor‐β; TNF‐α, tumor necrosis factor‐α

ELISA was used to detect the levels of TNF‐α, TGF‐β, IL‐6, and IL‐1β in the serum of MCAO rats, and also to determine the effects of casticin on inflammatory cytokines. As shown in Figure 5, after rats were treated with casticin for 7 days after MCAO modeling, serum levels of TNF‐α, TGF‐β, IL‐6, and IL‐1β in the MCAO group were significantly higher than those in the sham group, whereas the levels of these cytokines in the casticin 20 mg/kg and 40 mg/kg treatment groups were lower than those in the MCAO group (*p* < 0.05). The results indicated that the inflammatory cytokines TNF‐α, TGF‐β, IL‐6, and IL‐1β were involved in the pathological injury process of cerebral ischemia, whereas casticin could significantly reduce their expression and the damage to brain cells.

### 3.3 Casticin reduced apoptosis of brain cells in MCAO rats

**FIGURE 6:**
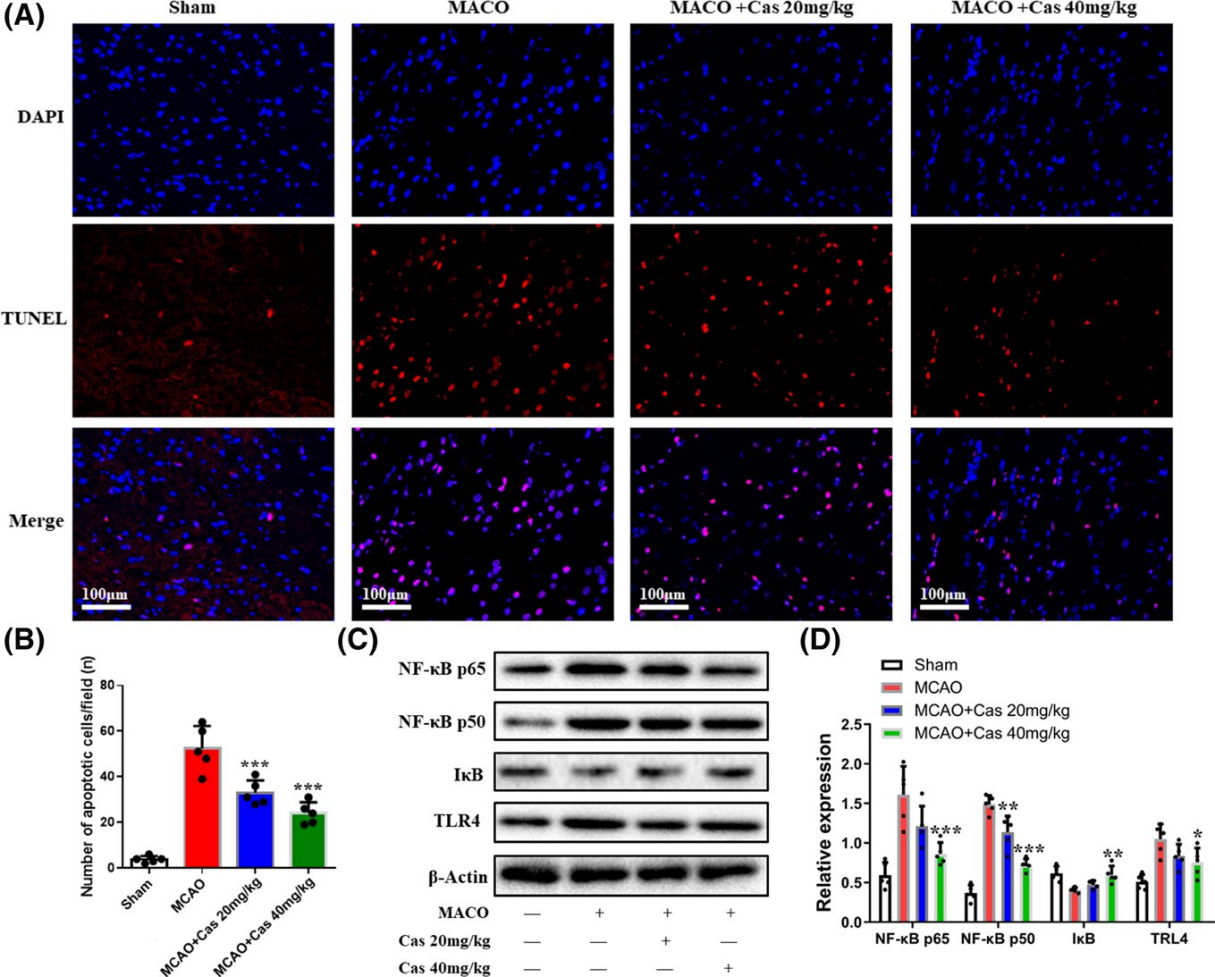
Apoptosis of brain cells in MCAO rats and the expression level of TLR4/NF‐κB signaling protein in brain tissue. (A‐B) Apoptosis of nerve cells detected by TUNEL. Red is RUNEL and blue is DAPI, scale bar =100 μm (n = 5); (C‐D): The expression levels of TLR4, NF‐κB p65, NF‐κB p50, and IκB in brain tissue of MCAO rats detected by Western blot. Each loading protein was 30 μg (n = 5). *p < 0.05, **p < 0.01, ***p < 0.001. DAPI, 4’,6‐diamidino‐2‐phenylindole; IκB, NF‐κB inhibitor protein; NF‐κB, nuclear factor‐κB; OGD/R, oxygen glucose deprivation/reperfusion; TLR4, Toll‐like receptor 4; TUNEL, TdT‐mediated dUTP‐biotin nick end labeling

The TUNEL method was used to detect apoptosis of cells in MCAO rat brains after the rats were treated with casticin for 7 days after MCAO modeling. Results showed that the apoptotic cell number (52.40 ± 9.91 cells/field) in the MCAO group was larger than that in the sham group (3.80 ± 1.48 cells/field). The 20 mg/kg and 40 mg/kg casticin groups had apoptotic cell numbers of 33.00 ± 5.43 and 24.00 ± 4.85 respectively, with less apoptosis than in the MCAO group (*p* < 0.05, Figure 6A‐B). These findings showed that cerebral ischemia could promote the apoptosis of brain cells, whereas casticin could reverse or inhibit the degree of apoptosis.

### 3.4 Casticin reduced the expression of TLR4/NF‐κB

TLR4‐induced inflammatory response of NF‐κB is closely related to the development of cerebral ischemic infarction.^21^ To investigate the potential mechanisms involved in the protective effect of casticin on neural functions in MCAO rats, we measured the expression levels of TLR4, NF‐κB p65, NF‐κB p50, and IκB proteins in brain tissue of MCAO rats. As shown in Figure 6C‐D, after the rats were treated with casticin for 7 days after MCAO modeling, the expression levels of TLR4, NF‐κB p65, and NF‐κB p50 in the MCAO group were significantly higher than those in the sham group, and the expression of IκB protein was significantly lower than that in the sham group. However, treatment with casticin (20 mg/kg or 40 mg/kg) significantly inhibited the expression of TLR4, NF‐κB p65, and NF‐κB p50 and elevated the expression of IκB (*p* < 0.05). The results indicated that casticin decreased the expression of TLR4, NF‐κB p65, and NF‐κB p50, and reversed the expression of IκB in cerebral ischemic tissue.

### 3.5 Casticin reduced apoptosis of OGD/R‐PC12 cell and inhibited the expression of TLR4/NF‐κB

**FIGURE 7:**
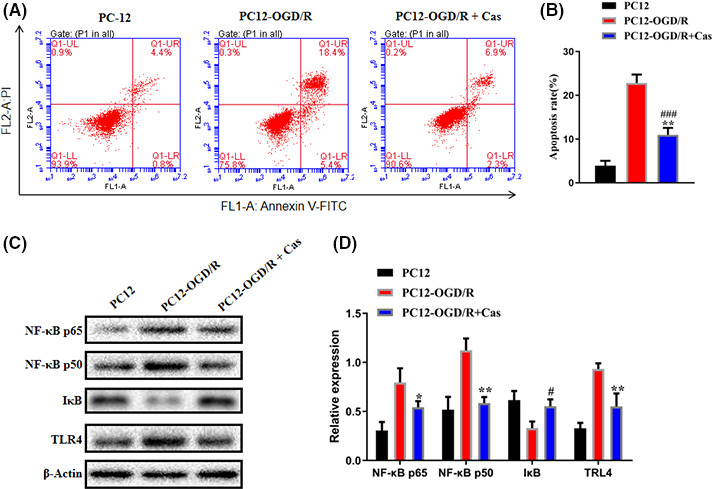
Effects of casticin on apoptosis of OGD/R‐PC12 cells and proteins in the TLR4/NF‐κB signaling pathway. (A‐B) The apoptosis of OGD/R‐PC12 cells after treated with casticin (10 μM) for 24 h; (C‐D): Western blot showing the expression levels of TLR4, NF‐κB p65, NF‐κB p50. and IκB after treatment with casticin (10 μM) for 24 h in OGD/R‐PC12 cells (n = 5). *p < 0.05, **p < 0.01

**FIGURE 8:**
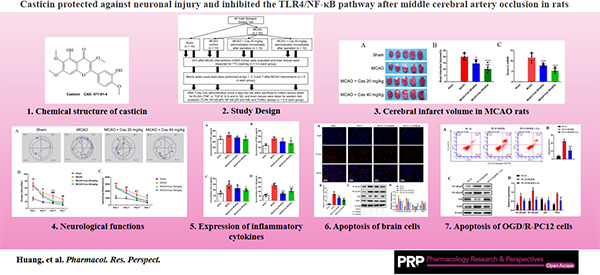
Research ideas of Casticin against neuronal injury

To further clarify the neuronal protective role of casticin, we used PC12 cells to establish an oxygen glucose deprivation/reoxygenation (OGD/R‐PC12) model. First, the degree of apoptosis of OGD/R‐PC12 cells was detected by flow cytometry. The results showed that the apoptosis rates were 22.83 ± 1.94 (%) and 10.97 ± 1.59 (%) (*p* < 0.05, Figure 7A‐B) in the OGD/R‐PC12 model group and OGD/R‐PC12 + 10 µM casticin groups, respectively. Similarly, we found that the expression levels of TLR4, NF‐κB p65, NF‐κB p50, and IκB proteins in the OGD/R‐PC12 model group was higher than those in control PC12 cells, and the expression of IκB protein was significantly lower than that in control PC12 cells. Casticin‐treated OGD/R‐PC12 cells exhibited decreased expression of TLR4, NF‐κB p65, and NF‐κB p50 and enhanced expression of IκB protein, which indicated that casticin may protect OGD/R‐PC12 cells by inhibiting the TLR4/NF‐B signaling pathway (Figure 8).

## 4 DISCUSSION

Flavonoids compounds are secondary metabolites in plant growth process and widely exist in fruits, vegetables, herbage, and medicinal plants. They are not only abundant in quantity, but also vary in structure, types, and quite a few of them have significant neuroprotective effects. Flavonoids compounds such as quercetin, wogonin, baicalein, catechin, epigallocatechin, and others can inhibit the expression of inducible nitric oxide synthase (iNOS) and cyclooxygenase 2 (COX2), reduce the production of NO and the release of cytokines, and inhibit the production of reactive oxygen species (ROS) in nerve cells, and thus play a role in regulating neuroinflammation.^22^, ^23^ In addition, flavonoid compounds are natural radical scavengers, such as baicalin which can decrease malondialdehyde (MDA) levels and increase the activity of superoxide dismutase (SOD) and glutathione (GSH) against oxidative stress in the hippocampus when transient cerebral ischemia occurs.^23^ 3'‐O‐methyl‐catechin has also been proved to block the c‐Jun‐N‐terminal kinase (JNK) signaling pathway and thus protect neurons from oxidative damage.^24^ Casticin, as a kind of flavonoid compounds, has been reported to inhibit acetylcholinesterase (AChE) and activate the ERK‐CREB‐BDNF signaling pathway as well as alleviate the cognitive dysfunction caused by cholinergic blockade.^23^ It can also inhibit the NF‐κB signaling pathway, thereby reducing arthritis and other inflammation associated to cartilage degeneration.^25^, ^26^ In addition, casticin can induce apoptosis in a variety of tumor cells.^27^ Casticin could dock to the protein crystal structure of PI3K to inhibit the activity of PI3K; casticin could form hydrogen bonds with PI3K at two amino acid sites, Val854 and Agr770, and a hydrophobic bond with PI3K at Val851. Thus, the PI3K/AKT pathway could be inhibited by casticin.^28^ NF‐κB is the downstream pathway of PI3K/AKT.^29^ In this study, the inhibition effect of casticin on the activation of NF‐κB pathway might be the results of casticin‐induced PI3K inhibition. In the present study, the TTC staining results revealed that casticin reduced the area of cerebral ischemia and infarction in MCAO rats. The mNSS scores and Morris water maze test results revealed that casticin had a neuroprotective effect on MCAO rats, and could also improve their neuronal functions, which suggested that casticin could be developed into a novel neuroprotective agent. However, casticin may interfere with the albumin‐binding of Site II ligands as well as the metabolism of drugs by acting on CYP2C9/CYP3A4 enzymes.^30^ CYP2C9/CYP3A4 inhibition is the limitation of castivin. Thus, the effective and safe dose showed and/or molecular modification of castivin should be performed to enhance its therapeutic role.

The inflammatory cascade reaction is an important mechanism of cerebral ischemia‐reperfusion injury.^31^ The expression levels of TNF‐α, TGF‐β, IL‐6 and IL‐1β secreted by microglial cells are vital in inflammatory cascade reactions. These factors can induce other inflammatory mediators, strengthen the adhesion of leukocytes and endothelial cells, promote the synthesis of nitric oxide, induce the generation of amino acid and free radical, and initiate multiple cascade reactions, which further leads to irreversible neuronal apoptosis in the brain region with cerebral ischemia.^32^, ^33^, ^34^ Recent studies have shown that casticin has a strong inhibitory effect on inflammation in DSS‐induced ulcerative colitis, lipopolysaccharide‐induced acute lung injury, and IL‐1‐induced human osteoarthritis.^13^, ^35^, ^36^ In the present study, results have demonstrated that casticin inhibited the expression of inflammatory cytokines TNF‐α, TGF‐β, IL‐6, and IL‐1β and reduced brain cell apoptosis. It was found that 40 mg/kg casticin had a better anti‐inflammatory cytokines effect than 20 mg/kg casticin. However, the expression of IL‐1β was slightly higher in 40 mk/kg casticin group compared with 20 mg/kg casticin group. Although, the inhibition effect of 20 mg/kg casticin on IL‐1β expression was better than that in 40 mg/kg group, both group showed efficient inhibitory role. As for therapeutic effect and functional recovery, 40 mg/kg casticin had better effect. Our *in vitro* studies also indicated that casticin could reduce the apoptosis of OGD/R‐PC12 cells.

TLR4 binding to its ligand activates myeloid differentiation factor 88 (MyD88), which transfers signals to the members of the IL‐1 receptor‐related kinase (IRAKs) family. After activation, IRAKs bind to tumor necrosis factor receptor‐associated factor 6 (TRAF‐6) to form a complex, which in turn activates transforming growth factor β kinase 1 (TAK‐1) via the TAK‐1 binding protein (TAB). It further activates NF‐κB‐induced kinase (NIK) and phosphorylates the NF‐κB inhibitor kinase (IKK) complex.^37^, ^38^ The activated IKK complex (IKKα and IKKβ) induces IκB ubiquitination and degradation, causes NF‐κB free activation, and translocates to the nucleus to bind to the promoter of inflammatory regulatory genes, which can promote transcription and synthesis of TNF‐α, TGF‐β, IL‐6, and IL‐1β and other inflammatory cytokines, thus initiating an inflammatory cascade.^39^, ^40^ Previous studies have reported that casticin had significant effects on NF‐κB, JNK, and MAPK,^41^, ^42^ but the effects of casticin on TLR4 remained unclear. We found that casticin significantly inhibited the expression of TLR4, NF‐κB p65, and NF‐κB p50 and promoted the expression of IκB protein in MCAO rats. Our *in vitro* results also revealed that casticin inhibited the TLR4/NF‐κB signaling pathway in OGD/R‐PC12 cells.

However, there were several limitations in this research. First, the study used MCAO rat model to clarify the treatment effect of casticin in brain stroke injury, there was a long way to translate the beneficial effect of casticin from rats to human, and more verification experiments on rats, monkey, and clinic trail should be done. Second, the accurate binding targets of casticin in stroke are needed to be identified in further studies.

## 5 CONCLUSIONS

This study indicates that casticin can reduce the size of the ischemic infarction, improve animal functional recovery and spatial learning ability in MCAO rats. Besides, we propose a potential mechanism of the neuroprotective effect of casticin as an inhibitor of the activity of the TLR4/NF‐κB signaling pathway, inhibition of the expression of inflammatory factors TNF‐α, TGF‐β, IL‐6, and IL‐1β, and thus weaken the inflammatory response after cerebral ischemia, leading to a reduction in neuronal apoptosis. The present findings provide the basis for future studies on casticin treatment for stroke‐induced brain injuries.

## ETHICS APPROVAL

The animal ethics committee of Hainan Medical University approved all experimental procedures conducted on rodents.

## CONFLICT OF INTEREST

The authors declare that they have no conflict of interest.

## AUTHOR CONTRIBUTIONS

Liu Q conceptualized and supervised the study; Huang D, Zhou JF, Li WN, Zhang L, and Wang XM designed and performed the experiments; Liu Q, Huang D, and Zhou JF interpreted results, and wrote the manuscript. All authors have read and approved the manuscript.

## Data Availability

The data that support the findings of this study are available from the corresponding author upon reasonable request.
